# Thermal Error Prediction and Compensation of Digital Twin Laser Cutting Based on T-XGBoost

**DOI:** 10.3390/s22187022

**Published:** 2022-09-16

**Authors:** Chang Lu, Jiyou Fei, Xiangzhong Meng, Yanshu Li, Zhibo Liu

**Affiliations:** 1 School of Mechanical Engineering, Dalian Jiaotong University, Dalian 116042, China; 2 Army Artillery and Air Defense Academy Sergeant School, Shenyang 110000, China; 3 College of Locomotive and Rolling Stock Engineering, Dalian Jiaotong University, Dalian 116042, China; 4 School of Mechanical and Electrical Engineering, Shanxi Datong University, Datong 037009, China

**Keywords:** time-varying, hybrid modeling, volumetric strain, T-XGBoost

## Abstract

Laser cutting belongs to non-contact processing, which is different from traditional turning and milling. In order to improve the machining accuracy of laser cutting, a thermal error prediction and dynamic compensation strategy for laser cutting is proposed. Based on the time-varying characteristics of the digital twin technology, a hybrid model combining the thermal elastic–plastic finite element (TEP-FEM) and T-XGBoost algorithms is established. The temperature field and thermal deformation under 12 common working conditions are simulated and analyzed with TEP-FEM. Real-time machining data obtained from TEP-FEM simulation is used in intelligent algorithms. Based on the XGBoost algorithm and the simulation data set as the training data set, a time-series-based segmentation algorithm (T-XGBoost) is proposed. This algorithm can reduce the maximum deformation at the slit by more than 45%. At the same time, by reducing the average volume strain under most working conditions, the lifting rate can reach 63% at the highest, and the machining result is obviously better than XGBoost. The strategy resolves the uncontrollable thermal deformation during cutting and provides theoretical solutions to the implementation of the intelligent operation strategies such as predictive machining and quality monitoring.

## 1. Introduction

There are significant improvements in processing new styles of metallic materials in many pillar industries, such as aerospace and transportation, attributable to the rapid development of the intelligent manufacturing. Meanwhile, the requirement of the accuracy and reliability of laser cutting is continuously increasing. As a representative of non-contact processing, the accuracy indicators of laser cutting are different from those of the traditional machine tools. The traditional NC machining is mainly turning and milling. Tool wear directly affects energy consumption [1], cutting force [2], surface quality of the workpiece [3], residual stress distribution of the workpiece surface [4], and production cost [5] during cutting. Different from the traditional machine tool, there are no errors caused by the induced force during the laser-cutting process. Instead, 70% of the errors are caused by thermal error during the laser-cutting process, which is the largest source of errors. Therefore, it is reasonable to conclude that the machine with a lower percentage of thermal errors has a higher accuracy [6,7,8].

There are a large number of in-depth studies on the thermal error of machine tools from domestic and international scholars, which mainly focused on the empirical thermal error model and the theoretical thermal error model [9]. The empirical thermal error model usually refers to the best processing parameters obtained through repeated processing and experiments. This method not only wastes a lot of manpower and material resources, but also has no reference value for other material processing. However, the limitations of empirical thermal error compensation methods are highlighted given that the operators cannot perform real-time manual error compensation during the automated CNC control process, which has been increasingly utilized in practice nowadays. Thermal errors in the processes can be accurately predicted and reasonably avoided by using theoretical error models. Zhao et al. [10] simulated the temperature field and deformation field of the machine tool spindle using the finite element (FEM) method and optimized the temperature sensitive point. Zhang et al. [11] took the whole machine tool as the research object and discussed the FEM thermal boundary conditions of the spindle system. Wu et al. [12] used FEM to model the thermal error of the screw feed system. FEM is widely used in the thermal error modeling of traditional contact machine tool processing. However, it is seldom used in non-contact cutting. The simulation accuracy is high when the boundary conditions are not complicated. However, the boundary conditions usually need to be re-tested and optimized and cannot be directly used for thermal error compensation [9].

Some scholars built thermal deformation prediction models by using machine learning. Fujishima et al. [13] proposed a new compensation method using a deep-learning algorithm to compensate for thermal deformation in the machine structure. Postel et al. [14] designed a method for inverse identification and prediction of parameters during cutting operations. Lin et al. [15] proposed a method to predict the transverse and tensile strength of SPR joints based on the XGBoost algorithm and verified that the prediction error was less than 7.6%. One of the advantages of using machine-learning algorithms to predict thermal deformation is that the model is scalable which means with more data from machine processing and algorithm optimization, the accuracy of the prediction can be improved accordingly. However, it is time consuming and expensive to obtain a large set of data to achieve a more accurate prediction. To solve this problem, Liu et al. [16] proposed a method based on FEM and artificial neural network (ANN) models for predicting and compensating deformations in machine tools with a two-machine riveting system. The machining accuracy is improved.

Currently, the research on thermal errors in machine tools is mainly focused on the contact machining, such as turning and milling. Dynamic optimization methods have mostly focused on tool wear detection for contact machining [17]. There is limited research on the thermal error in the field of non-contact machining, including laser cutting. The precision of the laser cutting is greatly affected by the machined parts being processed and the processing conditions. As a result, a FEM-model-driven solution or data-driven solution cannot meet the real-time requirements. Digital twinning technology realizes the accurate mapping of physical entities in digital space and promotes the interaction between laser cutting workshops and intelligent systems. With the help of the concept, this paper constructs the transmission of processing information between physical and virtual entities [18,19]. To establish a laser cutting thermal error prediction and compensation model based on the mapping ability of the digital twin technology to time-varying characteristics [13], it is necessary to consider the whole life cycle of processing [20]. It not only analyzes the changes in machine tool performance but also processing parameters in the time dimension. In addition, the prediction simulation before machining is added and the cutting process is further optimized by using the prediction information. Therefore, this paper proposes a prediction and compensation method for thermal deformation of laser cutting based on digital twin technology, beyond the existing thermal error compensation schemes for contact machining, which adopts the hybrid modeling techniques of “model-driven and data-driven”. It uses the thermo-elastic–plastic finite element (TEP-FEM) model to obtain real-time thermal deformation data and the prediction ability of the XGBoost algorithm to improve the accuracy of laser-cutting process parameters.

## 2. Materials and Methods

### 2.1. The Main Characteristics of Laser Cutting Accuracy

**Figure 1:**
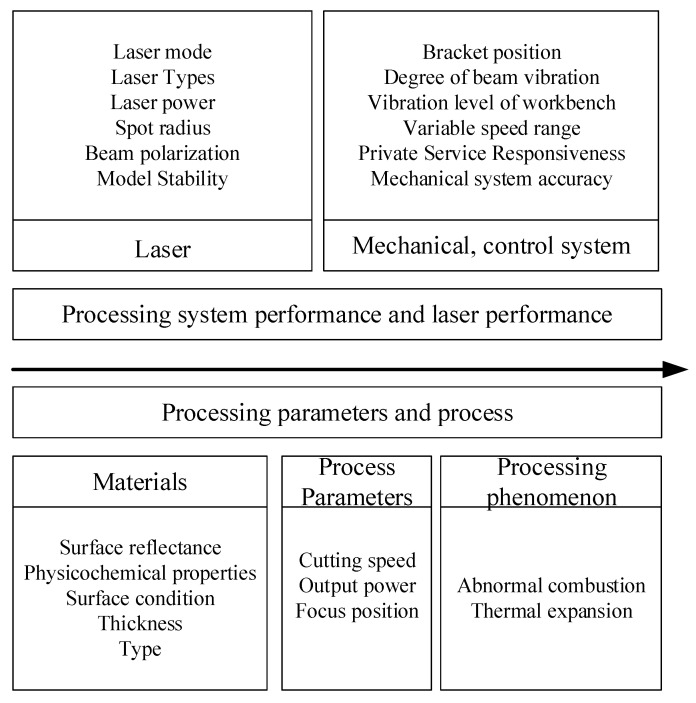
Factors affecting laser cutting accuracy.

Laser cutting is widely used due to its high speed and high precision. The overall precision of laser cutting is determined by the machine performance, laser performance, properties of the workpiece, processing parameters, and processing phenomena, see Figure 1. However, it is difficult to study the main characteristics of real-time machining accuracy based on the machine performance and the long decay period of the laser generator [21].

For a known type of material, the laser cutting accuracy is primarily determined by the processing parameters and the thermal phenomenon during the cutting process. In addition, the heat absorbed by the workpiece is impacted by the cutting speed. During the cutting process, the surface temperature field and the structure field of the workpiece form a solid–thermal coupling [4], resulting in thermal expansion. Therefore, the cutting speed is selected as the main characteristic for the variation in laser cutting accuracy.

### 2.2. Material

Laser cutting is commonly used to alloy materials. Beyond alloy material, it has been increasingly used on highly reflective materials with the development of laser cutting technology. In this paper, the cutting processes of three common materials in industry are analyzed, i.e., high-speed steel (W18Cr4V), carbon structural steel (Q235A), and 2A12 aluminum alloy, of which the 2A12 aluminum alloy is a highly reflective material and needs surface treatment. It is found in the preliminary research that the specific heat capacity and thermal conductivity of the material can impact the processing quality during the cutting process to certain extent, therefore, it is also considered as a variable factor in the modeling [22]. The physical properties of the material are shown in Table 1 [23].

### 2.3. Initial Conditions

The performance of the laser cutting machine can significantly impact the cutting accuracy. Therefore, the research group cooperated with Dalian Locomotive Technician College and used the highly precise machine, Swiss Bystronic BySmart Fiber3015 cutting machine, as the processing equipment, which is equipped with the Fiber 3000 laser. Table 2 below shows the actual parameters when cutting the three types of material selected as previously mentioned as initial data for modeling, each with four thicknesses that are commonly used in practice.

### 2.4. Hybrid Model Methods

Most of the existing laser cutting parameter optimization schemes are empirical models, which improve the cutting accuracy by adjusting the cutting speed based on a large amount of processing results. Although are they time-consuming and labor-intensive with high costs, those models also do not sufficiently consider the impact on the slit from the changes in the laser generator motion from time to time.

**Figure 2:**
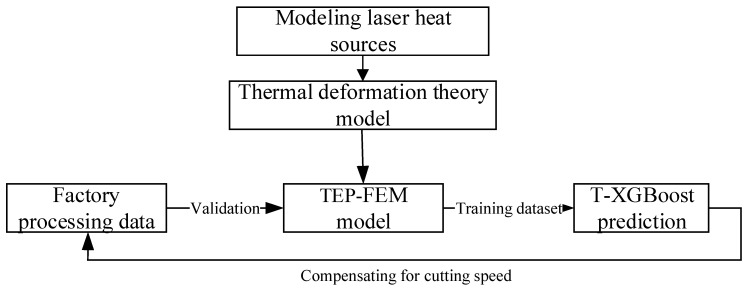
Hybrid model methods flow chart.

The cutting speed varies during the operation of the laser cutting machine, especially in the beginning and end stages. A full-cycle speed optimization strategy can be established utilizing the time-varying mapping capability of digital twin technology. The method steps are as follows, as shown in Figure 2:

1. Establish the laser heat source model;
2. Establish the thermal deformation theoretical model;
3. Establish a visual TEP-FEM simulation model and obtain real-time thermal deformation data;
4. Use the factory’s mature technical parameters to verify the rationality of the TEP-FEM model;
5. Use the TEP-FEM dataset as the training dataset on T-XGBoost to optimize the actual processing data;
6. The optimized thermal deformation is used as the offset to compensate the real-time error of the cutting speed.

Among them, the processing parameters used in step 4 are provided by Dalian Locomotive Technical College and the verification method is shown in reference [22].

As a result, the virtual cutting accuracy prediction and analysis is achieved through the high approximation simulation under the hybrid model of “model-driven and data-driven”, which is driven by the ability of transmission of virtual and real information introduced by the digital twin technology.

#### 2.4.1. Heat Source Model

The laser cutting equipment has a complex structure. During the machining process, there is no contact between the cutting equipment and the workpiece, except for the bracket fixing the workpiece. The impact on the simulation results of the rest of the complex structure of the laser cutting equipment is minimal. Therefore, it is reasonable to simplify the mechanical part as the laser heat source. The laser is not a uniform heat source and the equation of the heat flow density is:

(1) $$q\left(x,y\right)=q_{m}\cdot e^{-K\left(x^{2}+y^{2}\right)}$$

(2) $$q_{m}=\frac{P\cdot K}{\pi }$$

**Figure 3:**
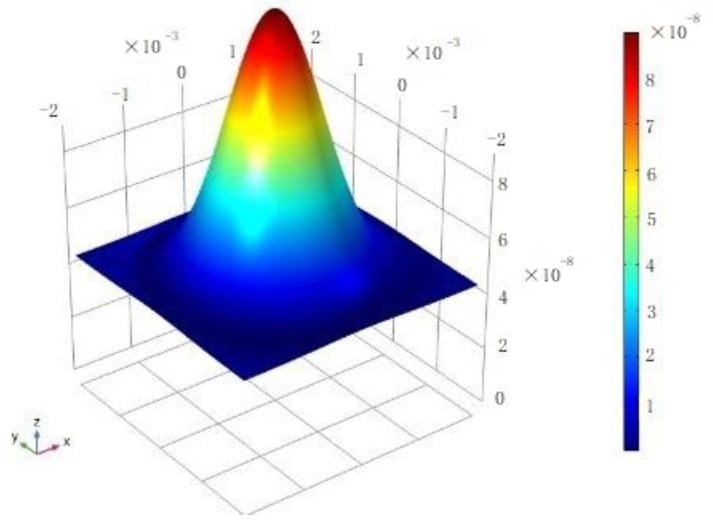
Heat source model.

$q_{m}$ is the maximum heat flow in the center of the heat source, P is the total power of the heat source, and K is the heat source concentration coefficient. The heat flow density at any point (*x*, *y*) is related to its distance from the central maximum heat source point. In another word, the closer to the central point, the higher the heat flow density. The margin of heat flow density is related to the heat source concentration factor. The heat source concentration is modeled with a power of 3000 W and a laser focus radius of 2 mm, as shown in Figure 3. The model is established by COMSOL 5.4. The research object within this paper is a thin steel plate, which is one of the primary workpiece types processed by laser cutting and widely used in the industry. Another major type of workpiece processed by laser cutting is steel pipe. However, the performance of cutting steel pipe is limited by the fixture and has no obvious advantages in high-speed processing.

#### 2.4.2. Finite Element Model

The thin steel plates using laser cutting technology are primarily thin-walled parts with thicknesses of 0.5~12 mm. It is appropriate to divide thin plate parts into triangular elements. Mesh refinement is performed at the slits to enhance the calculation accuracy and reduce the waste of material. The refinement is performed on the longest side of the workpiece since the slit will be much larger than the light spot during the cutting process with high speed. If the grid at the slit was larger than the laser spot radius, it would severely affect the simulation accuracy as the middle part would be skipped.

**Figure 4:**
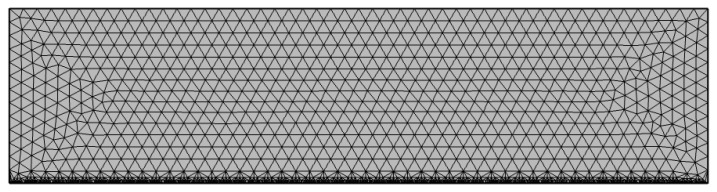
Encrypted FEM model at the slit.

The heat source model in Section 2.4.1 shows that the laser heat source is concentrated with a large temperature gradient. Therefore, it is necessary to refine the meshes on the moving path of the heat source to truly reflect the temperature change and deformation of the area affected by the laser heat source. The mesh at the slit is encrypted four times by mesh irrelevance verification, and the mesh division is shown in Figure 4.

In addition to the mesh model, the variation in material properties of the processed workpiece with temperature is introduced in the modeling process. The changes in thermal conductivity and specific heat capacity are embedded in the model, and the specific data are shown in Table 1.

#### 2.4.3. Boundary Conditions

The characteristics of the heat transfer in laser processing follow Fourier’s law, which includes three basic modes of heat conduction, heat convection, and heat radiation. Heat loss exists in every thermal phenomenon in the cutting process, and the boundary conditions are divided into three categories, as shown in Formula (3).

(3) $$\{\begin{array}{c} T{┃}_{\Gamma 1}=T\left(\Gamma ,t\right)\;\\ \vec{n}\cdot {\left(k\nabla T\right)}_{\Gamma 2}=q_{s}\left(\Gamma ,t\right)\\ -\vec{n}\cdot {\left(-k\nabla T\right)}_{\Gamma 3}=\varepsilon \sigma \left(T_{\mathrm{amb}}{}^{4}-T^{4}\right) \end{array}$$

The first type of boundary condition describes the temperature distribution on the system boundary, where *Γ* is the boundary range, *T* is the temperature, and t is the time. When $T{┃}_{\Gamma 1}$ is a constant, it is a steady state condition, and when *T*(*Γ*, *t*) is expressed as a time function, it is an unsteady heat source. The second type of boundary condition describes whether there is heat inflow or outflow on the boundary, where $q_{s}$ is the heat flux density, and $\vec{n}$ is the direction of the normal line outside the system boundary. When $\frac{\partial T}{\partial n}{┃}_{\Gamma 2}=0$, it is an adiabatic boundary; when $\frac{\partial T}{\partial n}{┃}_{\Gamma 2}$ is a constant, it is a constant heat flow boundary; when $\frac{\partial T}{\partial n}{┃}_{\Gamma 2}$ is a time-dependent function, it is a non-constant heat flow boundary. During the cutting process, the laser heat source moves along the cutting trajectory with the constant cutting speed, therefore, it is a non-stationary and non-constant heat flow boundary, which varies from time to time.

The whole machining process is simulated by the TEP-FEM simulation method. Considering three heat loss modes, heat conduction, heat convection, and heat radiation, complete boundary conditions are formulated to improve the simulation accuracy. The research of Gutiérrez G. et al. [24,25] mostly refers to the consideration of the thermal radiation mode but ignores the influence of thermal convection and thermal radiation. Although the influence of thermal convection and thermal radiation on deformation is less than that of thermal convection [26], when the machining accuracy reaches or exceeds the nanometer level, it still directly affects the yield. All should be considered in high-speed and high-precision cutting.

The third type of boundary condition describes the heat exchange between the system and the outside environment. k is the heat transfer coefficient, ε is the surface emissivity, σ is the Stefan–Boltzmann constant, and $T_{amb}$ is the ambient temperature. The heat absorptivity of the high-reflectivity material surface is low, and as a result it is the part with the largest heat loss during the laser-cutting process. Q235 has a better surface heat absorption rate of 70%, and the rate of W18Cr4V can also reach 60%. The absorption rate of 2A12 aluminum alloy is only about 10% before the surface refinement. If the surface is simply refined using the carbon ink with a good absorption rate, the absorption rate can increase to 42.32% [27]. The wind speed in the closed or semi-open workshop space is less than 0.15 m/s, which approximates the natural air heat transfer. The temperature variant during processing is large, but the heated area is small. The laminate material has a large area in contact with the air and has better heat dissipation performance. The air heat transfer coefficient is 10, and the processing ambient temperature is room temperature of 20 °C.

At present, most research only considers the influence of heat conduction but ignores the heat convection and heat radiation. For high-precision machining, any heat loss will affect the simulation accuracy. All three heat transfer conditions are considered in this model.

#### 2.4.4. Temperature Field

Based on the data in Section 2.2 and Section 2.3, the finite element model is established using the above method. By simulating the relative motion between the laser heat source and the workpiece, the heat transfer phenomenon in the cutting process is analyzed.

#### 2.4.5. Thermal Deformation 

During the laser-cutting process, the heat source moves at a high speed and heats the slit spot, causing the thermal expansion and elastic deformation at the slit, which lasts for a short time, and gradually recovers after the heat source passes through. When the thermal stress in the heated area exceeds the yield limit of the material, plastic deformation occurs, which has a significant impact on the machining accuracy [28,29]. Based on the temperature field analysis in Section 2.4.4, the solid–thermal method is carried out to analyze the deformation during the machining process.

#### 2.4.6. T-XGBoost Model

XGBoost, an emerging integrated learning algorithm, improves the prediction stability and accuracy by combining multiple weak learners with a strong learner. Ma et al. used the XGBoost algorithm to predict the classification and sensitivity for clay materials [30]. It was also verified that the prediction performance of the XGBoost is better than Artificial Neural Network (ANN) and Bayesian (NB), and this algorithm is considered more suitable for the engineering industry. Bae et al. used the XGBoost algorithm to predict solar photovoltaic power generation. They also compared the accuracy with that of the Long–Short Memory method (LSTM) and the Mean Error method (MAPE) and verified the unique advantages of the XGBoost algorithm for energy conversion [31]. It improves the performance of boosting models through pruning, parallelization, and term regularization by using the XGBoost algorithm and reduces the overfitting of the traditional decision tree algorithms. The XGBoost algorithm has been gradually applied in various fields [32], and currently is rarely used in the laser-cutting process. The XGBoost regression of the prediction value calculation [33,34] is shown in (4):

(4) $$\hat{y_{t}}=\sum_{k=1}^{K}f_{k}\left(x_{t}\right),f_{k}\in F$$

$\hat{y_{t}}$ represents the predicted value, $f_{k}$() represents the kth tree model, $x_{t}$ represents the input features, and t represents the number of trees, which represents the functional space consisting of a set of trees. The objective function in the XGBoost regressor includes a regularization term [28,29], as shown in (5):

(5) $$O_{bj}=\sum_{t=1}^{n}l\left(y_{t},\hat{y_{t}}\right)+\sum_{k=1}^{K}\Omega \left(f_{k}\right)$$

*l*() represents the mean square error (MSE) of the loss function, $y_{t}$ represents the actual value, and Ω() represents the regularization term that causes loss to the model complexity, as shown in Equation (6):

(6) $$\Omega \left(f\right)=\gamma T+\frac{1}{2}\lambda \sum_{j=1}^{T}\omega_{j}^{2}$$

where *T* represents the time point, $\omega_{j}$ represents the thermal deformation amount at the corresponding time point, and *γ* and *λ* represent the penalty factors.

**Figure 5:**
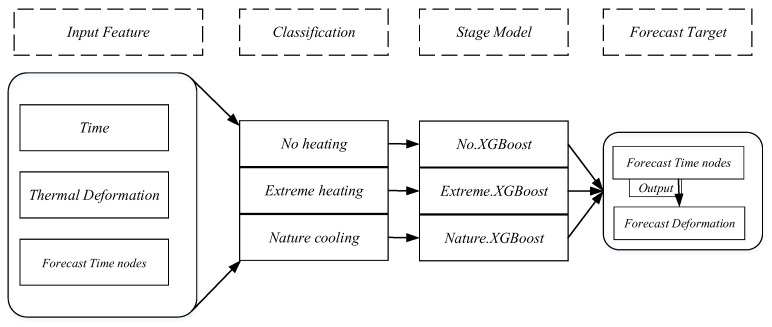
T-XGBoost thermal deformation prediction process.

Predictions are made using the XGBoost algorithm tree by utilizing the resulting branched data based on the input features. This paper proposes a segmentation model in the time series (T-XGBoost model). Given the target data can be from different stages, different prediction models can be established for each specific stage by using the tree structure of the XGBoost algorithm to combine the classification task and the regression task. The T-XGBoost model uses different training data sets for different stages of the prediction target. The booster parameter is set to gbtree, the model is a tree-based model, and hyperparameter tuning is performed for maximum depth, minimum weight, and subsamples. The termination conditions are set according to the machining accuracy requirements, and other parameters remain default. To improve the accuracy of the model and reduce the learning time, the thermal deformation in Section 2.4.5 is used as the training dataset for different stages. The prediction process is shown in Figure 5.

## 3. Results

### 3.1. Temperature Field Results

Based on the data in Section 2, TEP-FEM modeling simulations were used for the 12 working conditions to obtain the transient temperature clouds of the cutting process, the temperature variation curves at specific points, and maximum temperature variation curves of the machined part in the whole domain with time.

**Figure 6:**
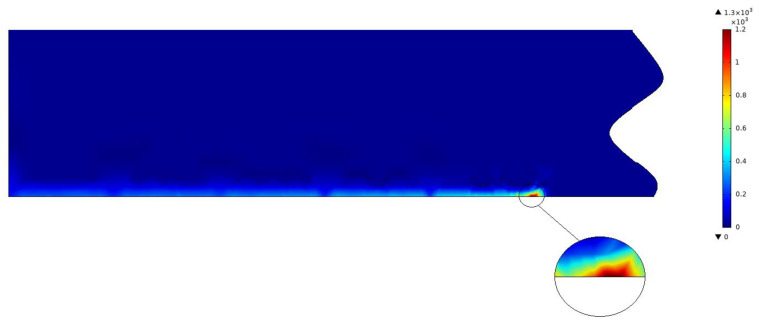
Transient temperature cloud at 1 s of W18Cr4V cutting.

**Figure 7:**
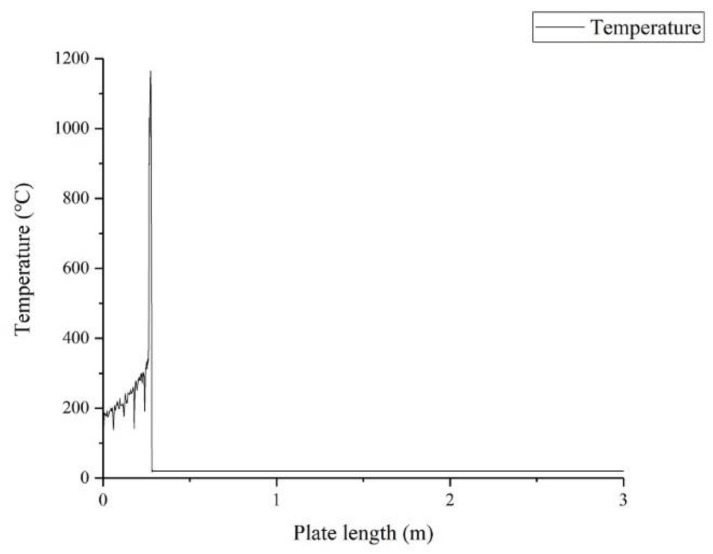
Temperature profile on the cut path at 1 s of W18Cr4V cutting.

**Figure 8:**
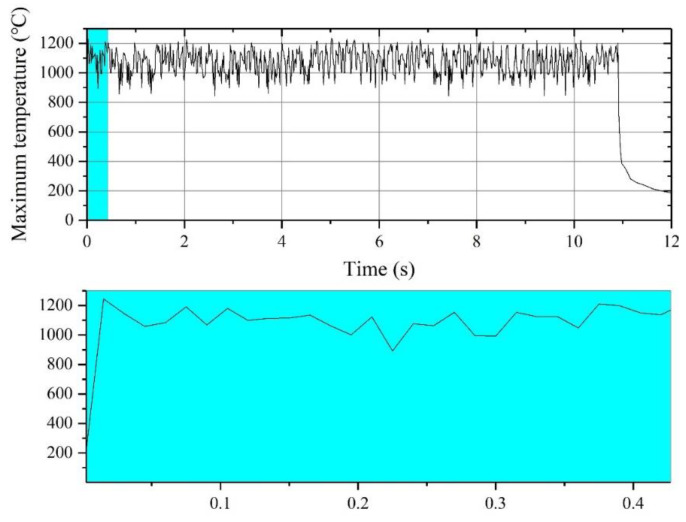
The curve of the maximum temperature at the W18Cr4V slit with time.

Take condition 2 as an example, Figure 6 shows the cloud diagram of the temperature field at a certain time of laser cutting. It is shown in Figure 7 that the temperature change curve is at the slit at this time. The highest temperature position corresponding to the laser focus position is 1185.5 °C. Figure 8 shows the maximum temperature value of the thin plate. At the beginning of cutting, the temperature at the cutting seam rises rapidly and reaches a stable value at 0.015 s. After stabilization, it fluctuates up and down around the maximum value, and the average stable temperature is 1183.5 °C. It is cooled naturally after cutting.

Using the data obtained above, the mean value of the extreme heating time and the stable temperature was calculated via logistic regression and statistical analysis based on the Levenberg Marquardt algorithm and the Gauss–Newton linear model (instead of a nonlinear function), as shown in Table 3.

Working conditions 1–4 use the same material W18Cr4V, and the thickness gradually increases. The time for condition 1 to reach stability is slightly longer than condition 2, which is 0.032 s. Under conditions 2–4, the stabilization time gradually becomes longer. The lowest stable temperature of the four working conditions is 1161.87 °C, and the highest is 1197.89 °C. The processed material is Q235 under working conditions 5–8. With the increase in the thickness, the time to reach the stable temperature increases, and the longest time is 0.84 s. The average stable temperature is close, all falling within the range of 1473.03 °C~1486.95 °C. The processed material is 2A12 aluminum alloy under working conditions 9–12. With the increase in the thickness, the stable temperature increases from 0.02 to 1.6 s. The stable temperature of working condition 9 is close to that of working condition 12, which is 676.36 and 674.94 °C. The stable temperature of working conditions 10 and 11 is slightly higher, reaching 762.72 and 747.68 °C.

### 3.2. Thermal Deformation Results

In order to better express the size of thermal deformation, volume strain is chosen to represent it. With the increase in temperature, the thermal conductivity and specific heat capacity of the material also change. Adding the change in related parameters to the material properties makes the simulation results of deformation more accurate.

**Figure 9:**
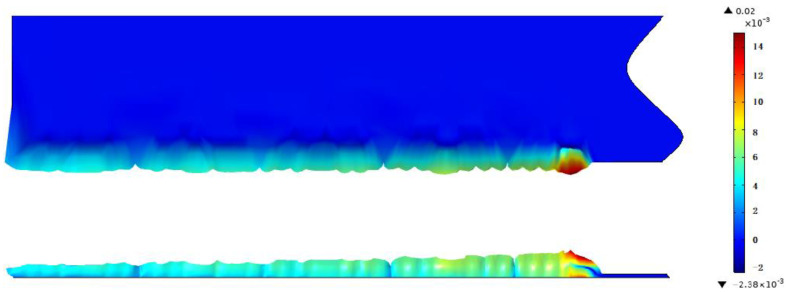
Thermal deformation of the slit when cutting for 1 s in working condition 2 (scale factor 1000).

Under working condition 2, for example, there is deformation at the slit, while the largest deformation occurs at the laser focus point. With the movement of the heat source, the area that has been cut is gradually cooled down, and the thermal expansion and elastic deformation is gradually recovered. After the material completely cools down, there is still some remaining irreversible plastic deformation, as shown in Figure 9.

Three specific points, i.e., the origin, 0.5 m, and 1 m are taken at the slit [35]. The volumetric strains at the origin of condition 1 is close to 0.5 m and 1 m, which are 1.3567, 1.3578, and 1.3522. In the other 11 working conditions, the volume strain at the origin is the smallest, and the other two points are close. When the processing material is fixed, the volume strain decreases with the increase in the thickness of the workpiece, as shown in Table 4.

### 3.3. T-XGBoost Compensation Results

The thermal deformation results predicted by the T-XGBoost algorithm in Section 2.4.6 are used in the FEM model for recalculation. The parameters of all 12 conditions are kept the same except for the compensation change in the cutting speed to the thermal deformation. To verify the effectiveness of the compensation of the thermal deformation prediction to the thermal error, we compared the maximum value and average value of the thermal deformation at the slit under each condition. The results are shown in Table 5.

Using the XGBoost algorithm directly to compensate the cutting speed can reduce the maximum deformation by more than 33%. However, the average deformation of 12 working conditions is higher than that before compensation. The average maximum deformation of working conditions 4 and 12 increased by more than 100%. The T-XGBoost algorithm segments data in advance for prediction and compensation. Compared with compensation, the maximum deformation of the 12 working conditions was improved, which can reduce the maximum deformation by more than 45%. At the same time, the average volume strain of 11 working conditions decreases, which can be increased by 63% at the highest.

## 4. Discussion

### 4.1. Temperature Field Analysis

The results show that the trend of data variation is consistent under 12 working conditions, except for the time to reach the stabilization temperature and the value of the stabilization temperature. The heat flux density at the laser focus position is the highest, and the temperature around it gradually decreases, which is in line with the Gaussian heat source distribution characteristics. After the high-speed laser passes through, the temperature on the processing path gradually cools down over time. It is shown in Figure 7 that the temperature at the focus point is the highest, of which the left side gradually cools down over time. The unprocessed part on the right is at room temperature, which is consistent with the actual processing. Figure 8 shows three phases within the trend of the maximum temperature value of the thin plate. Phase I represents a rapid heating process where the temperature rises rapidly over the heating time. Phase II shows that during the cutting process, once a stable temperature is reached, the maximum temperature value fluctuates up and down, which follows the principle of the generation of the pulsed laser. Phase III is the natural cooling process at the slit after the cutting is completed.

The average of the stable temperature for each of the 12 working conditions exceeds the melting point of the respective material. For the thin plates with the same thickness, the larger the material absorption rate, the shorter the time needed to reach the stability, resulting in a relatively better cutting accuracy, except for working condition 1. The stable temperature under working condition 1 is slightly lower than the melting temperature of the material, however, given the stable nature of W18Cr4V and that the thermal conductivity and specific heat capacity are not significantly affected by temperature, a stable cutting can be achieved when the cutting temperature is higher than the melting point. Under working conditions 2–8, the cutting accuracy is high as the stable temperature falls within the material melting temperature range. Among the last four conditions, only the stable temperature under condition 11 falls within the melting temperature range. Although cutting can be achieved under working condition 10, since the stable temperature is slightly higher than the melting range, it is very likely to cause a large amount of vaporization, sublimation, slag, and other phenomena at the cutting seam, which affects the processing quality. The 2A12 aluminum alloy is a material with low melting point and high reflectivity, which is more difficult to cut than the other two materials. The stable temperature under working conditions 9 and 12 is higher than the melting point of the material and slightly lower than the melting temperature. Because of the active properties of aluminum alloy, the melting point can be quickly reached. Therefore, the cutting can be achieved with better cutting accuracy only under these two conditions, which is consistent with the fact that aluminum alloy material is more difficult to cut in actual practice. The cutting accuracy for aluminum alloy material is also a hot topic within laser cutting research.

### 4.2. Thermal Deformation Analysis

When the heat source is close to those points, the material expands significantly and the volumetric strain reaches the maximum value. After the heat source moves away, the elastic deformation of the material at the spot gradually recovers, and the volumetric strain gradually decreases and stabilizes at the plastic deformation value.

The origin point is the beginning of the processing, of which the thermal deformation is affected by cutting speed and motion inertia of the laser generator and is affected differently compared to other points. This is because the heat source moves at a uniform speed without preheating, the heat flux density at the origin point is lower than other locations, and as a result, the volumetric strain value is slightly lower. The other two points have similar volume strain and the deformation after natural cooling down, when the machining accuracy is at or below micron level. For certain type of material, the volume strain decreases when the thickness of the steel plate increases. After multiplying by the thickness of the workpiece to obtain the volume strain, the amount of thermal deformation at the slit of the same material is similar, and as a result, a micron-level processing accuracy can be achieved. The thinner the workpiece, the lower the machining accuracy. Multiplied by the thickness of the workpiece, the accuracy under condition 1 is lower than other conditions. The deformation under condition 11 is larger than the other three conditions for 2A12 aluminum alloy material, and the cutting accuracy is relatively lower, which is consistent with the temperature field result.

### 4.3. T-XGBoost Compensation Analysis

The quality of the laser kerf is primarily determined by factors such as the hanging slag at the kerf and the waviness and roughness of the cutting surface. Using the XGBoost algorithm directly to compensate for the cutting speed can reduce the maximum deformation. However, the average deformation is higher than that before compensation, which indicates that the periodic ripple of the cutting surface is obvious at this time. The maximum deformation is significantly reduced with the compensation calculated by the XGBoost algorithm, but the average deformation is higher than the pre-compensation condition. It means that the periodic ripple of the cutting surface is obvious, and therefore, the expected compensation effectiveness is not achieved. The periodic corrugation on the laser cutting surface is a geometric feature between the machining accuracy and surface roughness, which seriously affects the quality of laser cutting. This is because of the time series in the input data. The pre-cutting and natural cooling stages are at the poles of the data set and at room temperature, but the deformation is different, which significantly affects the prediction results of the algorithm. According to the data analysis, T-XGBoost can reduce the maximum deformation at the slit by more than 45%. At the same time, by reducing the average volume strain under most working conditions, the lifting rate can reach 63% at the highest point, and the machining result is obviously better than XGBoost.

## 5. Conclusions

In this paper, a thermal error prediction and dynamic compensation strategy of digital twin laser cutting based on T-XGBoost is proposed. Combined with the actual situation of laser cutting thermal deformation prediction, the XGBoost algorithm is optimized. TEP-FEM is widely used in thermal error simulation of contact machining (such as turning) [3,4,5], but it is not used in thermal error research of laser cutting. The XGBoost algorithm has been used many times to predict practical engineering problems and performed well [19,20,21], but it has not been used in laser cutting. The main conclusions are as follows:

(1) A hybrid model combining TEP-FEM and the T-XGBoost algorithm is established for laser cutting, which drives the possibility of real-time mapping of thermal deformation.
(2) According to the real situation of laser cutting temperature change, a more targeted T-XGBoost algorithm with a better optimization effect is proposed. The thermal deformation at any moment is obtained using simulation and used as the training data set of the algorithm. Higher cutting accuracy can be achieved by appropriately adjusting the cutting speed under a specific parameter.
(3) TEP-FEM and the T-XGBoost algorithm are used to realize the data flow between “model-driven and data-driven”, and the predicted thermal deformation is used to compensate the processing speed to improve the laser cutting accuracy.

This strategy is remarkably advanced comparing to the traditional empirical model as it can be applied to a wide range of materials. In addition, it can also obtain larger datasets through visual finite element simulations. In the virtual environment, this strategy and model can be used to pre-analyze and judge the reasonableness of the processing parameters, which will further help save time and reduce costs, with the potential of wider application in manufacturing. It provides theoretical support and application guidance for the realization of intelligent operation strategies such as laser cutting predictive processing and quality monitoring.

Future research will focus on the testing of the product using the cutting tests and algorithm optimization, especially the use in actual processing. We will use a coordinate measuring machine (accuracy 0.1 um) to measure multiple cuts under the machining parameters based on the optimized data and verify the machining quality. At the same time, the algorithm can be further optimized based on the measurement results to be more suitable for the non-contact machining. The influence of surface absorbance of high reflectivity materials on cutting conditions will be optimized. We will focus on the speed control and the use of related sensors.

## Figures and Tables

**Table 1:** Material properties.

Parameters	Thermal Conductivity λ W/(m*K)		Melting Point °C	Melting Temperature °C	Specific Heat Capacity Cp c/J·(kg·K)^−1^	
W18Cr4V	20 °C	27.21	1050	1180~1220	50 °C	473.11
	200 °C	25.96			200 °C	494.04
	500 °C	25.96			600 °C	457.81
	700 °C	25.12			900 °C	487.12
Q235A	200 °C	61.1	1468	1460~1495	200 °C	745
	300 °C	55.3			300 °C	770
	500 °C	42.7			400 °C	783
	700 °C	34.2			500 °C	833
2A12 aluminum alloy	121 (T4 Status)		659.8	720~760	100 °C	921
					200 °C	1047
					300 °C	1130
					350 °C	1172

**Table 2:** Initial processing parameters.

Work Condition	Material	Thickness mm	Laser Power W	Cutting Speed m/min	Pulse Frequency Hz	Focus Position mm
1	W18Cr4V	1	3000	36	1000	2
2		2	3000	16.5	1000	2
3		5	3000	2.9	1200	5
4		10	3000	0.5	1200	10
5	Q235	1	3000	33	1000	2
6		2	3000	15	1000	2
7		5	2400	2.8	500	5
8		10	2900	1.5	500	10
9	2A12 aluminum alloy	1	3000	19	2000	2
10		2	3000	8	2000	2
11		5	3000	2.6	2000	5
12		10	3000	0.9	2000	10

**Table 3:** Results of the temperature analysis for different working conditions.

Working Condition	Material	Thickness mm	Extreme Heating Time (s)	Stabilized Temperature Average (°C)
1	W18Cr4V	1	0.032	1161.87
2		2	0.015	1183.5
3		5	0.35	1194.98
4		10	1.08	1197.89
5	Q235	1	0.01	1476.45
6		2	0.1	1473.03
7		5	0.49	1468.84
8		10	0.84	1486.95
9	2A12 aluminum alloy	1	0.02	676.36
10		2	0.12	762.72
11		5	0.28	747.68
12		10	1.6	674.94

**Table 4:** Thermal deformation analysis results of different working conditions.

Working Condition	Material	Thickness mm	Volume Strain ‰		
			Origin	0.5 m	1 m
1	W18Cr4V	1	1.3567	1.3578	1.3522
2		2	0.7513	0.873	0.7912
3		5	0.1726	0.3846	0.351
4		10	0.021363	0.1024	0.09748
5	Q235	1	2.4324	2.577	2.4625
6		2	0.8255	1.0508	1.0272
7		5	0.1617	0.5762	0.4994
8		10	0.12676	0.2115	0.2222
9	2A12 aluminum alloy	1	1.0821	1.3068	1.2818
10		2	0.6917	1.051	0.9856
11		5	0.2353	0.5742	0.507
12		10	0.0993	0.1289	0.1161

**Table 5:** Compensation results under different working conditions.

Working Condition	Without Compensation (Volume Strain ‰)		XGBoost Compensation (Volume Strain ‰)				T-XGBoost Compensation (Volume Strain ‰)			
	Maximum Deformation	Average Deformation	Maximum Deformation	Lift Rate	Average Deformation	Lift Rate	Maximum Deformation	Lift Rate	Average Deformation	Lift Rate
1	4.56	1.3545	3.053	33%	1.38	−2%	2.508	45%	1.206	11%
2	10.39	0.8323	3.122	70%	0.94	−13%	1.898	82%	0.809	3%
3	9.472	0.3676	3.966	58%	0.592	−61%	3.987	58%	0.382	−4%
4	8.30	0.09994	3.356	60%	0.235	−135%	3.46	58%	0.0982	2%
5	16.596	2.51973	8.87	47%	3.121	−24%	7.086	57%	2.439	3%
6	21.73	1.0394	8.927	59%	1.892	−82%	9.063	58%	0.893	14%
7	13.47	0.53783	5.001	63%	0.898	−67%	5.768	57%	0.534	1%
8	13.696	0.21658	5.112	63%	0.307	−42%	5.71	58%	0.0796	63%
9	19.758	1.2943	8.199	59%	2.034	−57%	8.34	58%	1.0232	21%
10	25.123	1.0183	9.345	63%	1.431	−41%	10.52	58%	0.934	8%
11	18.411	0.5406	7.216	61%	0.79	−46%	7.696	58%	0.536	1%
12	11.146	0.1225	4.748	57%	0.262	−114%	4.674	58%	0.12	2%

## Data Availability

All data used to support the findings of this study are included within the article.
